# Structural insights into *Escherichia coli* polymyxin B resistance protein D with X-ray crystallography and small-angle X-ray scattering

**DOI:** 10.1186/s12900-014-0024-y

**Published:** 2014-12-05

**Authors:** Hunho Jo, Eui Young Jeong, Jinseong Jeon, Changill Ban

**Affiliations:** Department of Chemistry, Pohang University of Science and Technology, 77, Cheongam-Ro, Nam-Gu, Pohang, Gyeongbuk 790-784 South Korea

**Keywords:** PmrD, *E. coli*, SAXS, Crystal structure, Solution structure, Mutational study

## Abstract

**Background:**

Polymyxin B resistance protein D (PmrD) plays a key role in the polymyxin B-resistance pathway, as it is the signaling protein that can act as a specific connecter between PmrA/PmrB and PhoP/PhoQ. We conducted structural analysis to characterize *Escherichia coli* (*E. coli*) PmrD, which exhibits different features compared with PmrD in other bacteria.

**Results:**

The X-ray crystal structure of *E. coli* PmrD was determined at a 2.00 Å resolution, revealing novel information such as the unambiguous secondary structures of the protein and the presence of a disulfide bond. Furthermore, various assays such as native gel electrophoresis, surface plasmon resonance (SPR), size-exclusion chromatography, dynamic light scattering (DLS), and small-angle X-ray scattering (SAXS) measurements, were performed to elucidate the structural and functional role of the internal disulfide bond in *E. coli* PmrD.

**Conclusions:**

The structural characteristics of *E. coli* PmrD were clearly identified via diverse techniques. The findings help explain the different protective mechanism of *E. coli* compared to other Gram-negative bacteria.

**Electronic supplementary material:**

The online version of this article (doi:10.1186/s12900-014-0024-y) contains supplementary material, which is available to authorized users.

## Background

Various antibiotics have been widely used to inhibit bacterial growth or to kill bacteria. Polymyxin B, isolated from the bacterium *Paenibacillus polymyxa*, is one of the most potent antibiotics. It binds to lipopolysaccharide (LPS), the major component of the outer membrane of Gram-negative bacteria [1]. Because Gram-negative bacteria display the negatively charged LPS on the surface of the outer membrane, the cationic polymyxin B can interact with phospholipids to make the membrane permeable, resulting in the acceleration of water uptake, membrane disruption, and endotoxin release. It acts as a detergent against most Gram-negative bacteria, with the exception of the *Proteus* group [2-4].

Gram-negative bacteria have developed different protective mechanisms to counteract the effects of polymyxin B. For instance, *Salmonella enterica* (*S. enterica*) possesses a pair of two-component systems (TCSs) that are composed of PmrA/PmrB and PhoP/PhoQ. The TCSs are known to modify the acyl chain group or the phosphate group of LPSs, depending on environmental conditions. There are two types of pathways that activate the PmrA/PmrB system of polymyxin B resistance. The first pathway transpires during the growth of bacteria in the presence of high Fe^3+^ ion or mild acid pH and does not require PhoP/PhoQ [5], only the histidine kinase PmrB. The phosphorylation of PmrA by PmrB leads to the transcription of PmrA-activated genes, resulting in the modification of LPS layers. The second pathway includes the PhoQ/PhoP system and PmrD in conditions of low extracytoplasmic Mg^2+^ ions. PhoP is phosphorylated by the sensor PhoQ protein; phosphorylated PhoP then promotes the transcription of PmrD, a signaling protein that can act as a specific connecter between PmrA/PmrB and PhoP/PhoQ [6,7]. PmrD binds to the N-terminal domain of PmrA to prevent the dephosphorylation of PmrA, followed by the activation of the LPS modification process. However, the PmrD protein of *E. coli* does not play a role as a post-translational activator; thereby, the LPS modification only occurs via the first pathway in *E. coli*. It is expected to have different functions and structural characteristics compared with the PmrD protein in other bacteria such as *S. enterica* and *Klebsiella pneumoniae* (*KP*). Because the PmrD protein is affiliated to a new class of proteins that utilize different mechanisms of action for signal integration of bacterial cellular processes than those found in other signaling proteins, the identification of its structure and function is vital for a comprehensive understanding of TCSs systems in bacteria [8]. Therefore, the study of *E. coli* PmrD has been considered a decisive factor for determining discrepancies among several species [9-12].

In this study, we focus on exploring the structural features of *E. coli* PmrD. The findings reveal the crystal structure of *E. coli* PmrD in high resolution, and the structural traits are described in detail. Diverse assays were performed to determine the role of the internal disulfide bond. In addition, the solution structures of wild-type and mutant PmrD were determined using SAXS to obtain structural information in the solution environment.

## Methods

### Preparation of the proteins

The *E. coli pmrD* gene [UniProt: P37590] was isolated from K-12 genomic DNA and amplified by polymerase chain reaction (PCR) with specific primers. The amplified genes were inserted into the bacterial expression vector pET-15b (Novagen, Germany) containing the TEV protease cleavage site [12]. In addition, the C9A mutant was generated by a point mutation to eliminate the internal disulfide bond of PmrD. The His-tagged wild-type and mutant PmrD were expressed in the *E. coli* strain BL21(DE3) with Luria-Bertani broth containing 100 μg/L ampicillin, and were grown at 37°C to an OD_600_ of ~0.6. The expression of soluble PmrD was induced by the addition of 0.1 mM 1-thio-β-D-galactoside (IPTG), and the mixture was incubated in a shaker for 6 h. The cell lysate was treated with lysozyme in 20 mM phosphate buffer (pH 7.4) containing 500 mM NaCl and 2 mM β-mercaptoethanol. After sonication, the lysate was centrifuged at 13,000 rpm at 4°C for the removal of cell debris, and the resulting supernatant was loaded onto a Ni-chelating Sepharose fast-flow column charged with Ni ions (GE Healthcare Life Science, Sweden). The contaminants were washed with a washing buffer containing 20 mM phosphate (pH 7.5), 500 mM NaCl, 45 mM imidazole, and 2 mM β-mercaptoethanol. Wild-type and mutant PmrD were then eluted with washing buffer supplemented with 300 mM imidazole. The wild-type and mutant His-tagged PmrD were digested by adding TEV protease at 20°C for 8 h. To remove the uncleaved His-tagged proteins, the cleaved His-tagged fragments were reloaded for Ni-NTA affinity chromatography, and the target proteins were collected in the flow-through. For further purification, the eluted fractions were concentrated using Ultracel concentrators (Amicon) with a YM-10 membrane and loaded onto a HL Superdex 75 column (GE Healthcare Life Science, Sweden) equilibrated with 20 mM Tris (pH 7.0), 150 mM NaCl, 0.5 mM DTT, and 0.5 mM EDTA. Highly purified wild-type and mutant PmrD were concentrated to approximately 7–15 mg/mL by centrifugation and analyzed by 18% SDS-PAGE. In addition, uncleaved His-tagged wild-type and mutant forms were prepared for SPR measurement. The SeMet-substituted PmrD expressed in *E. coli* B834 (DE3) was purified via the same methods for native PmrD [12].

For the binding assay with PmrA, the *E. coli pmrA* gene [UniProt: P30843] was isolated from K-12 genomic DNA and amplified by PCR with specific primers, and was then inserted into the bacterial expression vector pET-28a (Novagen, Germany) containing the TEV protease cleavage site. The recombinant PmrA was overexpressed in the *E. coli* strain BL21(DE3) induced by 0.1 mM IPTG at 37°C, and purified as described above. The purity of PmrA was proven via gel electrophoresis.

### Protein crystallization and data acquisition

Crystals of PmrD were produced via the hanging-drop vapor-diffusion method at 20°C using 24-well Linbro plates (Hampton Research). The initial crystals were obtained after a period of one week at 20°C in 2.0 M ammonium sulfate and 50 mM sodium acetate (pH 4.6). Triclinic crystals were grown for one week to the maximum dimensions of 0.35 × 0.35 × 0.2 mm^3^. SeMet-substituted crystals were obtained in the same conditions. The crystals were transferred to a cryo-protectant solution and were flash-frozen in a nitrogen stream at 100 K. X-ray crystallographic diffraction data were collected with an ADSC Quantum 210 charge-coupled device (CCD) camera as the detector from the Beamline 4A of the Pohang Accelerator Laboratory (PAL) in Korea. The data for native PmrD and SeMet-substituted PmrD were collected, integrated, and scaled using HKL2000 software [13]. The data collection and processing statistics are summarized in Additional file 1: Table S1.

### Structural determination and refinement

Using the NMR structure of *E. coli* PmrD [PDB: 2JSO] as the starting model [14], phasing was performed via molecular replacement using MOLREP [15] in the CCP4 suite. All model building was achieved with COOT [16] and refined with REFMAC [17] and PHENIX software [18]. Water picking was accomplished with PHENIX using default parameters. The refined models were validated with PROCHECK [19]. All figures for the structural model were made in PyMol [20]. The structure-refinement statistics are also summarized in Additional file 1: Table S1.

### Accession numbers

The coordinate and structure factors for *E. coli* PmrD have been deposited in the RCSB Protein Data Bank (PDB), accession code 4HN7.

### Biochemical assays for the verification of the role of disulfide bridge in PmrD

To investigate the role of the internal disulfide bridge in PmrD, the binding affinities of wild-type and mutant PmrD with PmrA were estimated via native gel electrophoresis. Sample solutions with various ratios of PmrD to PmrA were prepared in 20 μL of reaction buffer (20 mM Tris–HCl, pH 7.8, 5 mM MgCl_2_, and 100 mM KCl) and incubated at 4°C for 1 h. Samples were loaded onto a 4% native polyacrylamide gel that was pre-run at 12 V/cm for 20 min, and they were analyzed at room temperature at 12 V/cm for 80 min in 1X TBE buffer. In addition, the functional change of PmrD caused by the disruption of the intra-disulfide bond was verified thoroughly using a Biacore T100 biosensor system (GE Healthcare, Sweden). A nickel solution was treated on the NTA sensor chip (GE Healthcare, Sweden), and then the His-tagged wild-type and mutant PmrD were separately immobilized on the chip in the running buffer (10 mM HEPES, 150 mM NaCl, 50 μM EDTA, 0.005% Tween 20, and pH 7.4). PmrA proteins with various concentrations ranging from 0.1 to 5 μM were applied to the PmrD-modified channel under continuous flow of 20 μL/min at 25°C. All SPR signals were assessed by a Biacore T100 response unit. The resulting data were analyzed using BIAevaluation software (v3.0), and the sensorgrams were reconstructed and examined using Origin 8.0.

The average sizes of wild-type and mutant PmrD were determined using size-exclusion chromatography. Based on a high-performance liquid chromatography AKTA system (GE Healthcare Life Science, Sweden), wild-type and mutant PmrD were loaded onto the HL Superdex 75 column (GE Healthcare Life Science, Sweden). The separation was carried out at 4°C at a flow rate of 1 mL/min with 25 mM HEPES (pH 7.0), 150 mM NaCl, and 5% glycerol as the mobile phase, and was monitored at an absorbance of 280 nm. Additionally, DLS is also beneficial to evaluate the size of target molecules. Each sample was prepared in solution containing 25 mM HEPES (pH 7.0), 150 mM NaCl, and 5% glycerol, followed by measurement in triplicate utilizing a Zetasizer Nano Z (Malvern Instruments, Malvern, UK). The average size distributions of the two types of PmrD are represented in Additional file 2: Figure S1.

### Determining solution-structure using SAXS

SAXS measurements were performed at the 4C beamline of the PAL. A light source from an in-vacuum undulator 20 (IVU 20: 1.4 m length, 20 mm period) of the Pohang Light Source II (PLS storage II) ring was focused on the sample, utilizing a vertical focusing toroidal mirror coated with rhodium and monochromatized with an Si (III) double crystal monochromator (DCM), giving an X-ray beam of wavelength 1.110 Å. The detector was a Rayonix 2D SX165, a two dimensional (2D) charge-coupled detector (Evanston, IL, USA). The sample-to-detector distance for SAXS was 2.0 m. The scattering angle was calibrated with poly(styrene-b-polyethylene-b-polybutadiene-b-polystyrene) block copolymer standards. The solution sample cell with mica windows was 10 μm thick with a volume of 50 μL, and it had an X-ray beam path length of 0.7 mm. All scattering measurements were conducted in the sample buffer containing 25 mM HEPES (pH 7.0) and 150 mM NaCl at 25°C. Because the scattering pattern was closely correlated with radiation damage, the SAXS data were collected in two successive timeframes of 30 s each, to monitor radiation damage. The absence of changes in the scattering patterns over time was confirmed, indicating that no radiation damage occurred during the scattering measurements. SAXS measurements were performed on protein solutions over the concentration range 1–15 mg/mL to obtain good-quality scattering data without any interference between the protein molecules (i.e., to eliminate any concentration effect). Scattering data were collected for 30 s. Each 2D scattering pattern obtained was circularly averaged from the beam center, normalized to the transmitted X-ray beam intensity, and corrected for scattering arising from the buffer solvent [21].

### SAXS data analysis

The experimental scattering patterns of SAXS were analyzed with a scattering program GNOM [22] to calculate the pair distance distribution function *p*(*r*) curve, expressed as follows:

$$ p(r) = \frac{1}{2{\pi}^2}{\displaystyle \underset{0}{\overset{\infty }{\int }}}qrI(q) \sin (qr)dq $$

where *q* is the magnitude of the scattering vector defined by *q* = (4*π*/*λ*)sin*θ*, 2*θ* is the scattering angle, *λ* is the wavelength of the X-ray beam, and *r* is the distance between the paired scattering elements in the protein. The radius of gyration from a Guinier plot (*R*_*g,G*_) can be calculated by fitting the measured scattering data with the Guinier equation [23], expressed as follows:

$$ I(q)=I(0) \exp \left[-\frac{q^2{R}_G^2}{3}\right] $$

The radius of gyration can be computed by a substitute method based on the *p*(*r*) curve. From the *p*(*r*) function, not only I(0) from the full scattering curve but also the maximum diameter of a given protein (*D*_*max*_) can be obtained from the distance at which *p(r)* approaches zero. *R*_*g,p(r)*_ is expressed by the following equation:

$$ {R}_{g,p(r)}^2=\frac{{\displaystyle \int }{r}^2p(r)\ dr}{2{\displaystyle \int }p(r)\ dr} $$

### Structural modeling and comparison among wild-type, mutant PmrD, and the crystal structure

The *ab initio* model-determination program GASBOR [24] was used to reconstruct the models of wild-type and mutant *E. coli* PmrD in solution. The reconstructed models were obtained without imposing any symmetry restrictions, and were averaged using DAMAVER [25]. The atomic coordinates of the crystallographic model of PmrD from the crystal structure [PDB: 4HN7] were converted into the SAXS curve using the CRYSOL program [26]. The SUPCOMB program was used to superpose the crystal structure onto the SAXS models reconstructed from the experimental data [27].

## Results and discussion

### Overall crystal structure of *E. coli* PmrD

**Figure 1:**
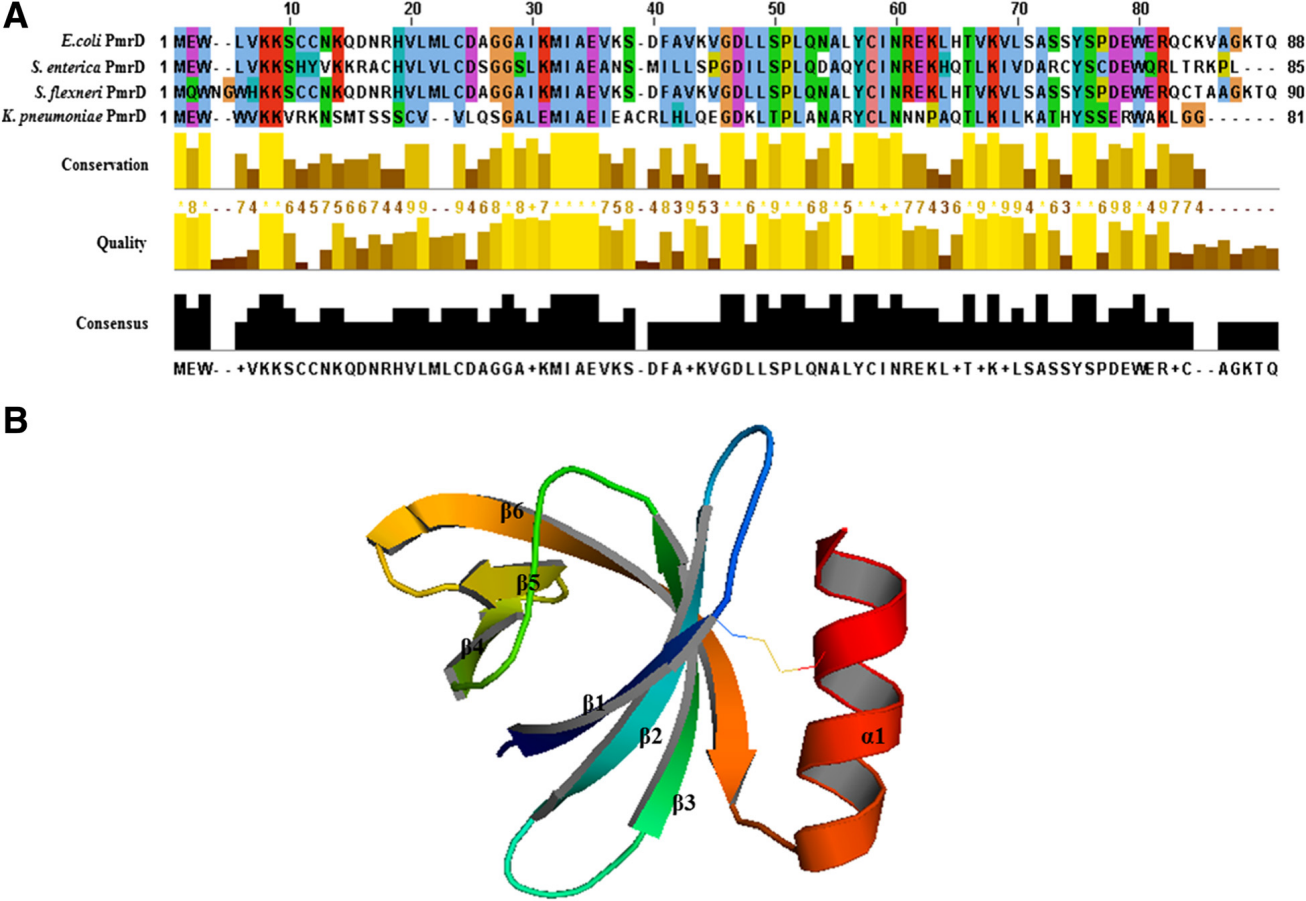
**Sequence alignment and crystal structure of** ***E*** **.** ***coli*** **PmrD. (A)** Sequence alignment between the PmrD proteins of *E. coli* and other bacteria. The results of the sequence alignment are represented as conservation, quality, and consensus using the Clustal Omega program. All sequences were obtained from NCBI. (*E. coli* PmrD, NP_416762.2; *S. enterica* PmrD, NP_456847.1; *Shigella flexneri* PmrD, NP_708145.1; *KP*-PmrD, YP_002237594.1). **(B)** Crystal structure of *E. coli* PmrD. The overall structure of *E. coli* PmrD is exhibited as a ribbon model. The secondary structures are indicated by the one α-helix (α1), six β-strands (β1, β2, β3, β4, β5, and β6), and loops in the ribbon model.

Prior to elucidation of the protein structure of *E. coli* PmrD, its amino acid sequences were compared with those of other species. As shown in Figure 1A, the amino acid sequences are highly conserved and their overall structures are coterminous. However, there are slight differences in the N-terminal (alignment number, approximately 10–18) and C-terminal (alignment number, approximately 83–88) regions. Structural determination was carried out to clarify the discrepancies in PmrD among various species.

**Figure 2:**
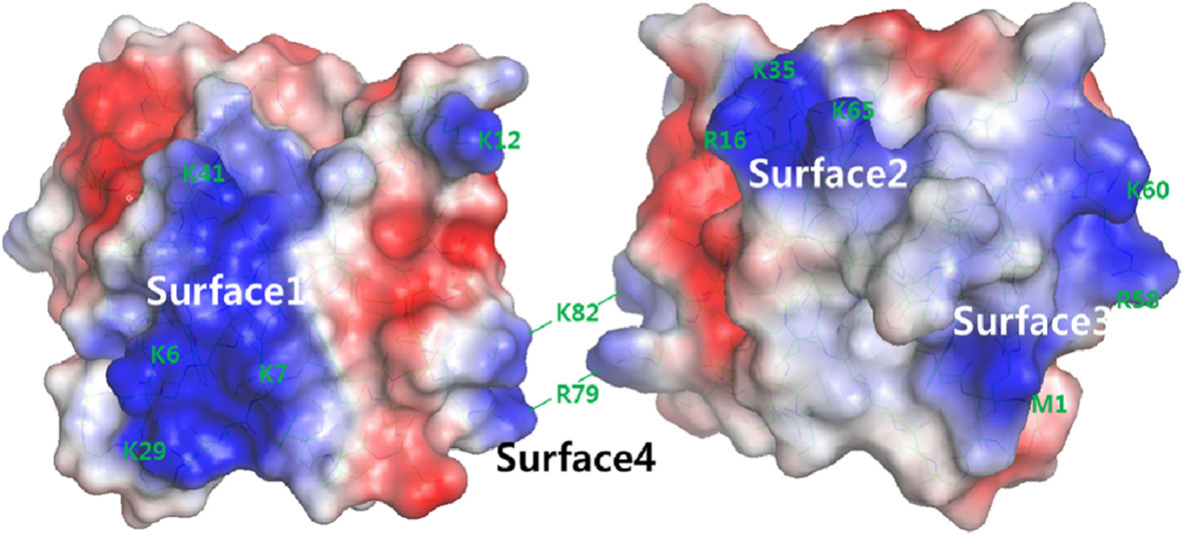
**The electrostatic potential of** ***E*** **.** ***coli*** **PmrD.** Surface illustration of *E. coli* PmrD colored by the electrostatic potential. There are four electropositive regions in the PmrD monomer (surface1, surface2, surface3, and surface4).

The crystal structure of *E. coli* PmrD was initially solved using the molecular-replacement method with the starting model, the NMR structure of *E. coli* PmrD [PDB: 2JSO]. According to the electron-density map, 88 residues were generally high quality, whereas the C-terminal region of the data was unclear. With the refinement program (REFMAC, CCP4i suite), the final structure of PmrD was refined at 2.00 Å so that the R_work_ and R_free_ values between the coordinates and the structure factors were 21.1% and 25.4%, respectively. The crystal structure of *E. coli* PmrD [PDB: 4HN7] was composed of a C-terminal α-helix and an anti-parallel β-barrel containing six β-strands (Figure 1B). The secondary structures of PmrD were defined via the MOLMOL [28] program (β1: Trp3-Lys7; β2: Arg16-Asp23; β3: Lys29-Lys35; β4: Leu45-Pro48; β5: Leu53-Ile56; β6: Glu59-Ser71; α-helix: Pro74-Ala84). Four long β-strands (β1, β2, β3, and β6) are located near the α-helix, and two short β-strands (β4 and β5) are situated on the opposite side of the α-helix. In addition, the surface electrostatic potential representations of *E. coli* PmrD were constructed using the PyMOL visualization program (Figure 2). The findings show many charged regions, including electropositive surfaces (surface1: Lys6, Lys7, Lys29, and Lys41; surface2: Arg16, Lys35, and Lys65; surface3: Met1, Arg58 and Lys60; and surface4: Arg79 and Lys82). Among these electropositive regions, surface1 was charged broadly, and surface2 and surface3 were located near each other. It can be expected that the surface1 or surface2-surface3 regions might interact with the electronegative region of PmrA (the phosphorylation site, Asp51) to inhibit the dephosphorylation of PmrA.

### Comparison of the *E. coli* PmrD crystal structure with other structures

**Figure 3:**
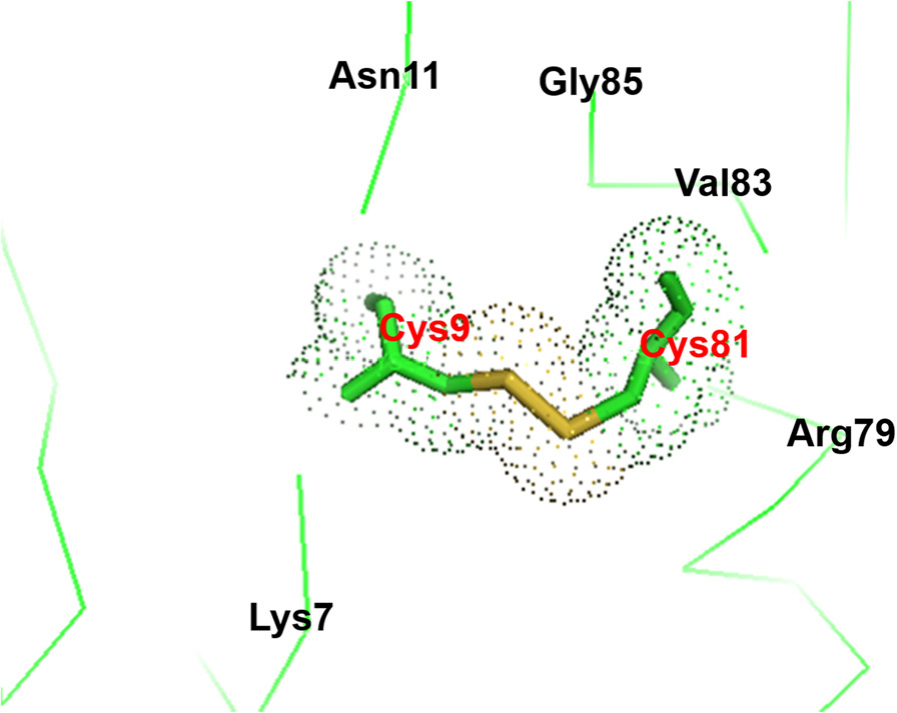
**Disulfide bond of** ***E*** **.** ***coli*** **PmrD between Cys9 residue in the β1 strand and Cys81 residue in the α-helix.**

The X-ray crystal structure of *E. coli* PmrD has numerous similarities to the NMR structure of the proteins, including six β-strands, an anti-parallel β-barrel with the topology of 6-3-2-1-4-5-6, and Greek-key topology. These characteristics also exist in *KP*-PmrD [29]. However, the PmrD proteins display some distinctions, such as the length of each strand, the definitude of structures, and the electrostatic potentials. In particular, the crystal structure exhibits well-defined secondary structures and one disulfide bond, in contrast to the *E. coli* NMR structure. The structure shows a disulfide bond between the Cys9 residue in the β1 strand and the Cys81 residue in the α-helix (Figure 3). The difference between our structure and that of the NMR is highlighted by factors such as the presence of several reducing agents, and the presence of the disulfide bond is confirmed by further biochemical experiments. This disulfide bond might contribute to the stabilization and fixation of the α-helix within the overall structure.

**Figure 4:**
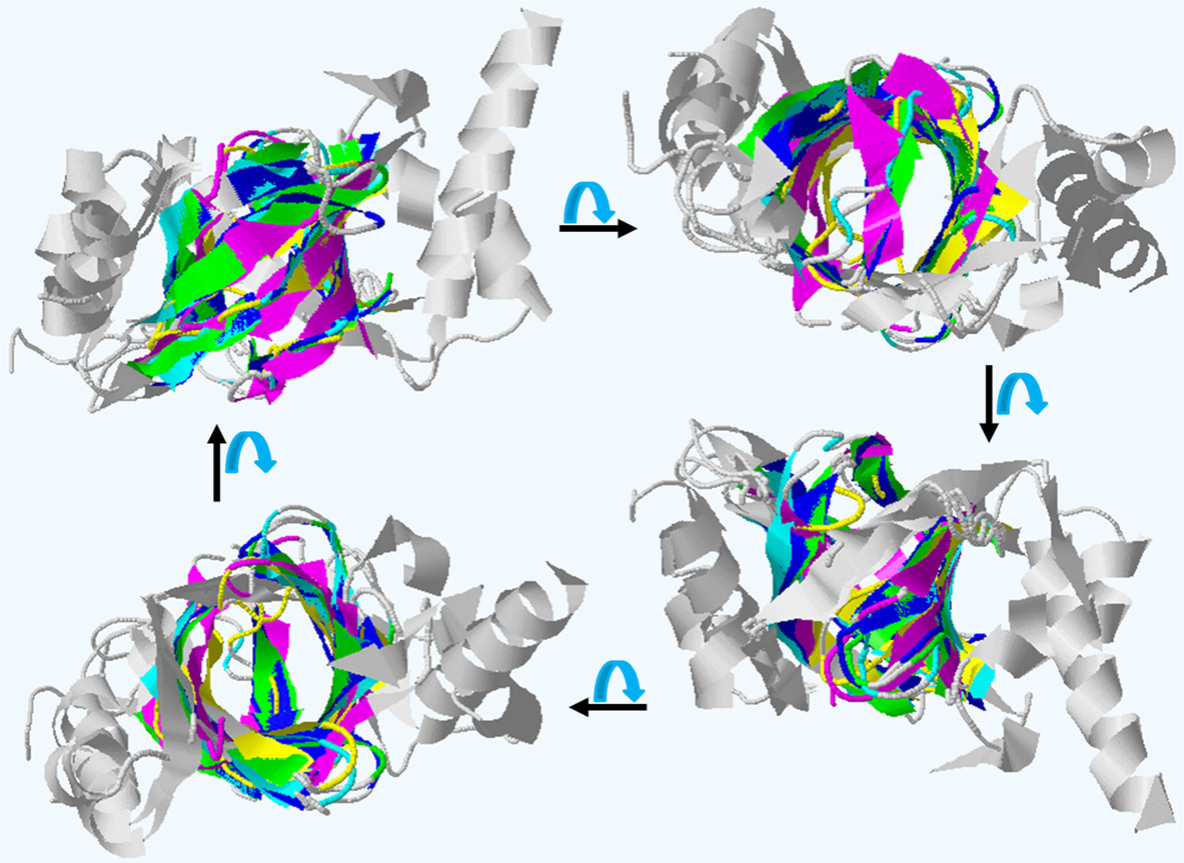
**Rotation view of the superimposed models using the PDBe Fold program.** The superimposed models were rotated at an angle of 90° on the horizontal axis and represented with the Jmol viewer (Yellowish-green, the crystal structure of *E. coli* PmrD: 4HN7; Blue, the NMR structure of *E. coli* PmrD: 2JSO; Sky-blue, the NMR structure of *KP*-PmrD: 2RQX; Yellow, the crystal structure of *T. kodakaraensis* HypC: 2Z1C; Wine, the crystal structure of excretory-secretory protein 2: 3NSW).

In accordance with the results of PDBe Fold (version 2.51) [30], ten PDB structures were found with Z-scores over 4.0 for the crystal structure of PmrD. Some models with Z-scores over 4.7 that shared a β-barrel structure containing several β-strands were superimposed onto the PmrD structure, as shown in Figure 4. The fold of *E. coli* PmrD belongs to the OB-fold domain, a conserved domain in bacteria and archaea. The OB-fold proteins with a five-stranded beta barrel structure can bind to nucleotides, proteins, and ions, and also have a special fold-related binding face (the center of β2 and β3), depending on the length and sequence of loops 2 and 4 [31]. The crystal structure of excretory-secretory protein 2 has two intra-disulfide bonds, one of which contributes to the stability of the C-terminal region in the same way as in *E. coli* PmrD [32].

### Verification of the functional change of mutant PmrD lacking in the intra-disulfide bond

**Figure 5:**
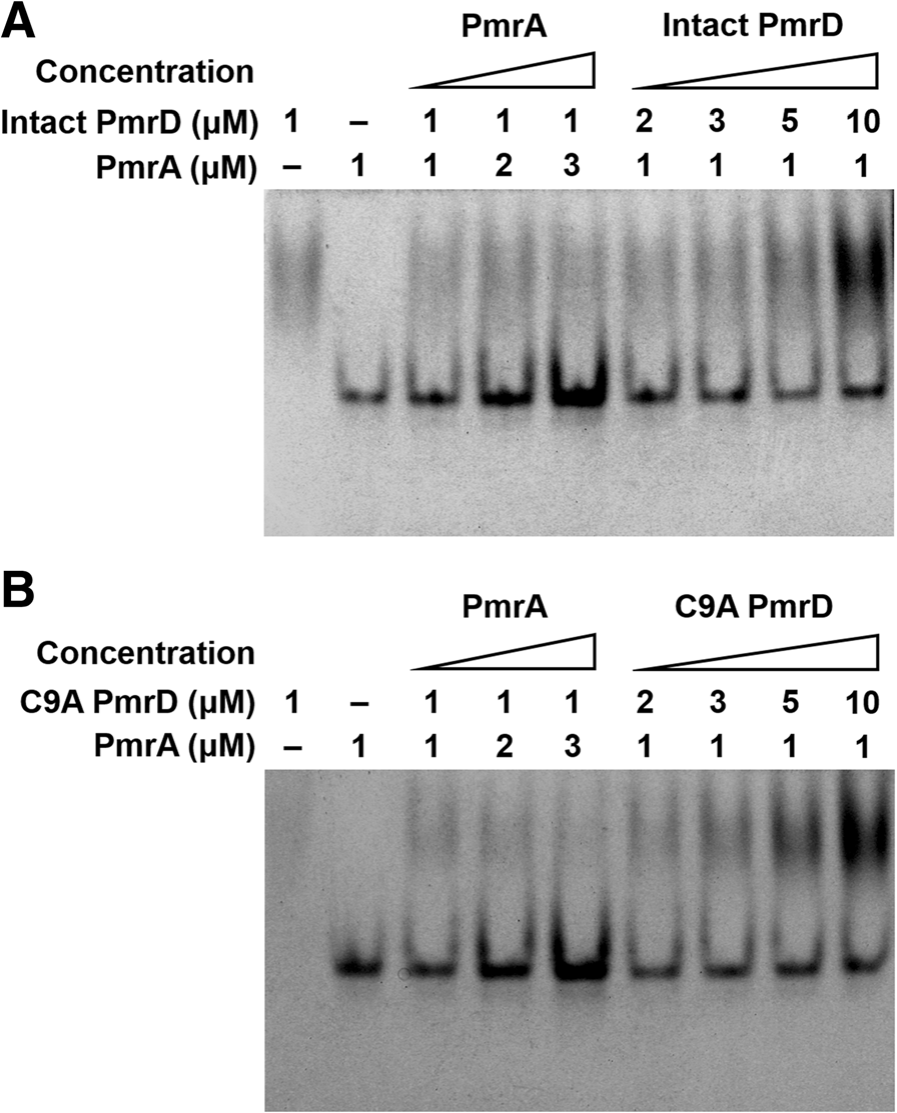
**Native gel electrophoresis for verifying the binding of wild-type and mutant PmrD with PmrA.** Sample solutions with various ratios of PmrD to PmrA were prepared in 20 μL of reaction buffer (20 mM Tris–HCl, pH 7.8, 5 mM MgCl_2_, and 100 mM KCl) and incubated at 4°C for 1 h. The samples were loaded onto a 4% native polyacrylamide gel that was pre-run at 12 V/cm for 20 min, and they were analyzed at room temperature at 12 V/cm for 80 min in 1X TBE buffer. The gels were stained with Coomassie Blue. **(A)** Wild-type PmrD. **(B)** Mutant PmrD.

To reveal the role of the disulfide bond in PmrD, C9A-mutated PmrD was generated by point mutation, resulting in the removal of the disulfide bridge. Prior to determining the structural disparity, the alteration of the PmrD function in the TCS, especially binding with PmrA, was validated through native gel electrophoresis and the SPR measurement. Because the lack of interaction between PmrD and PmrA inhibits the second pathway for the activation of the LPS modification process, the functional change of mutant PmrD is highly significant to the investigation of LPS modification in *E. coli*. For the gel analysis, highly purified PmrD and C9A PmrD were incubated with recombinant *E. coli* PmrA. As represented in Figure 5A, although the ratio of PmrA to wild-type PmrD was changed from 1 to 3, there was no multimer band in the gel. The higher ratio of wild-type PmrD did not affect the binding of wild-type PmrD with PmrA, in accordance with the previous report that *E. coli* PmrD did not interact with PmrA [14]. Elimination of the intra-disulfide bond in PmrD showed no distinct differences in the interaction of PmrD and PmrA, suggesting that the internal disulfide bond does not play a major role in binding with PmrA to prevent the dephosphorylation of PmrA (Figure 5B).

**Figure 6:**
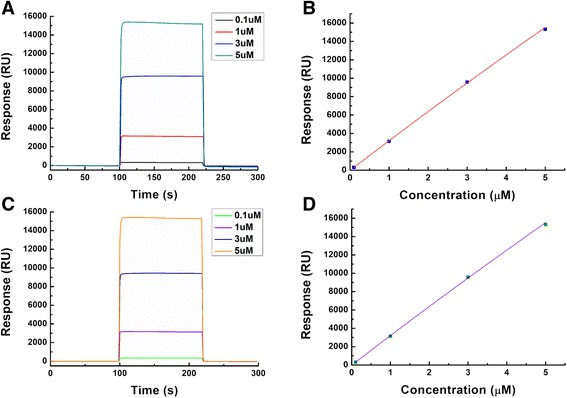
**SPR for the measurement of the binding strength between PmrD and PmrA.** The His-tagged wild-type and mutant PmrD were separately immobilized onto the NTA sensor chip in the running buffer (10 mM HEPES, 150 mM NaCl, 50 μM EDTA, 0.005% Tween 20, and pH 7.4). PmrA proteins with various concentrations (0.1, 1, 3, and 5 μM) were applied to a PmrD-modified channel under a continuous flow of 20 μL/min at 25°C using the Biacore T100 biosensor system. **(A)** Binding kinetics for wild-type PmrD with PmrA. **(B)** Plot based on the steady-state method for binding between wild-type PmrD and PmrA. **(C)** Binding kinetics for mutant PmrD with PmrA. **(D)** Plot based on the steady-state method for binding between mutant PmrD and PmrA.

The functional change in C9A PmrD was further demonstrated in detail, based on the SPR technique. After the His-tagged wild-type PmrD or mutant PmrD was immobilized onto the NTA chip by a coordinate covalent bond, triplicate injections of PmrA were performed at concentrations of 0.1, 1, 3, and 5 μM. The response signals at equilibrium following the injections were utilized to plot the response versus the PmrA concentrations to obtain the dissociation constant (K_d_). In the case of wild-type PmrD, PmrA exhibited a low binding affinity to wild-type PmrD as expected, with resulting difficulty in calculating the K_d_ value (Figure 6A and 6B). Based on the steady-state method: If K_d_ is larger than half of the highest concentration in the response-versus-concentrations graph, it cannot be calculated from the data. The same outcome was also observed in the C9A PmrD result, as represented in Figure 6C and 6D. There was no significant interaction between PmrA and mutant PmrD, indicating that the internal disulfide bond does not contribute to the function of PmrD.

### Confirmation of the structural variation of C9A PmrD

In the crystal structure of *E. coli* PmrD, it has been shown that the intra-disulfide bond may influence the maintenance of the overall structure. To judge the structural discrepancy between wild-type and mutant PmrD, specific experiments were conducted, including size-exclusion chromatography and DLS. Wild-type and C9A PmrD passed through the HL Superdex 75 column (GE Healthcare Life Science, Sweden). An analogous facet was observed in the profiles that displayed aggregation (~42 mL), dimer (~70 mL), and monomer (~80 mL) peaks. In particular, the elution volumes of the wild-type and mutant monomers were 82.014 mL and 82.000 mL, respectively, implying that their sizes obtained by size-exclusion chromatography were indistinguishable.

The DLS method is known to offer relatively accurate information on the size distribution profile in solution, and was therefore used to more precisely ascertain the size difference between wild-type and mutant PmrD. Through the triplicate measurements, the average sizes of wild-type and mutant PmrD were calculated as 3.668 ± 0.069 nm and 3.870 ± 0.057 nm, respectively. As exhibited in Additional file 2: Figure S1, the size of mutant PmrD at the maximum peak is larger than that of the wild-type form, and the overall distribution of C9A PmrD tends toward large particle sizes, indicating that the exclusion of the intra-disulfide bond in PmrD induces an increase in the overall size. This suggests that the disulfide bond might play an important role in the stabilization and fixation of the α-helix within PmrD.

### Reconstruction and evaluation of the solution structures of PmrD based on SAXS

SAXS is a valuable technique for analyzing the entire shape and size of target molecules in solution, and can provide more accurate size estimation than that achieved by size-exclusion chromatography or DLS. We introduced this method to clarify the structural transition of mutant PmrD lacking the intra-disulfide bond. The scattering patterns for *E. coli* PmrD were obtained from the 4C beamline at PAL. The measurements were taken at room temperature at 2.0 m from the CCD detector. Additional file 3: Figure S2 represents the SAXS profiles of wild-type and mutant PmrD measured in the sample buffer containing 25 mM HEPES (pH 7.0) and 150 mM NaCl. Additionally, the scattering data were contrasted with the theoretical SAXS profile evaluated from the PmrD crystal structure. All scattering patterns were similar, and the residuals of each scattering pattern displayed no systematic errors (Additional file 4: Figure S3). The Guinier plots and residuals were constructed from the scattering curve (Additional file 5: Figure S4). The radius of gyration (*R*_g_,_G_) values and the scattering patterns show no dependence on the concentration of the protein, implying that there is no interparticle interaction between the proteins. The plots exhibit linear characteristics in the Guinier *q* regions (*q* = 1.3*R*_g_, globular protein) and offer structural information to estimate *R*_g_,_G_. The calculated *R*_g_,_G_ values of wild-type and mutant PmrD were 14.10 Å and 14.63 Å, respectively. Additionally, C values were calculated by the below equation to demonstrate the availability of data in the range from 0.2-0.3 Å^−1^.

$$ \mathrm{R}<C=\frac{2\pi }{d_{max}}lo{g}_2\left(1+\frac{S}{N}\right) $$

The S/N ratios were 3.0534 and 2.4870, and the calculated C values were 0.2950 and 0.246137 for wild-type and mutant PmrD, respectively. Therefore, the data range from 0.2 to 0.3 Å^−1^ is acceptable.

Although the Guinier method is expedient to derive the radius of the target particles, it is influenced by several factors. Compared with the computationally straightforward Guinier function, the pair distance distribution function (*p*(*r*)) is comparatively unaffected by various factors such as the residual intermolecular interactions and the formation of aggregation, because it employs the entire scattering curve. Therefore, the maximum particle dimension (*D*_max_) and the radius of gyration (*R*_g_,_*p*(*r*)_) were derived from the *p*(*r*) function displayed in Additional file 6: Figure S5A (*D*_max_: 43.1 Å, *R*_g_,_*p*(*r*)_: 14.04 ± 0.02 for wild-type PmrD; *D*_max_: 46.0 Å, *R*_g_,_*p*(*r*)_: 14.46 ± 0.02 for mutant PmrD). Each *q*_min_ and *q*_max_ value was also presented (*q*_min_: 0.035, *q*_max_: 0.3317 for wild-type PmrD; *q*_min_: 0.035, *q*_max_: 0.3317 for mutant PmrD). All values from the Guinier plots and pair distance distribution functions are listed in Additional file 7: Table S2. The constructed *p(r)* functions present single peak patterns, which is characteristic of compact globular particles. The globular shapes of wild-type PmrD and C9A PmrD were also substantiated via the Kratky plots as shown in Additional file 6: Figure S5B. The similarity between *R*_g_,_G_ and *R*_g_,_*p*(*r*)_ indicates that the real space structure is precisely estimated from the scattering profile. In addition, the differences in *D*_max_ and *R*_g_,_*p*(*r*)_ between wild-type PmrD and C9A PmrD apparently suggest that the intra-disulfide bond in *E. coli* PmrD functions as a supporting force for the overall structure.

**Figure 7:**
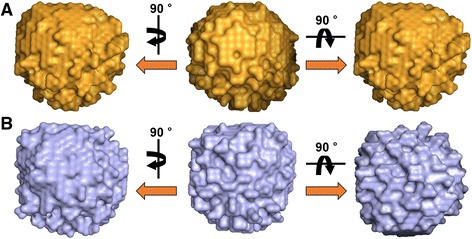
***Ab initio*** **models reconstructed from SAXS data using GASBOR.** The SAXS models are shown as sphere models. **(A)** Wild-type PmrD. **(B)** Mutant PmrD.

Because the *p*(*r*) function is an expression of the scattering profile in real space, it can be used to visualize target molecules. The *ab initio* SAXS model-independent structural models of wild-type and mutant *E. coli* PmrD were reconstructed using the GASBOR [24] and DAMAVER [25] programs, utilizing the GNOM results as a starting file. Based on specific information within the GNOM file, the GASBOR program established a model via a simulated annealing strategy. The 3D representations of proteins were acquired without assigning any restrictions on the symmetry or anisometry of the molecules. Each SAXS model was predicted 12 times and averaged via DAMAVER. Figure 7 shows the reconstructed solution structures of wild-type and mutant PmrD. Both have similar spherical structures, making it difficult to ascertain structural inequalities with only the figures.

**Figure 8:**
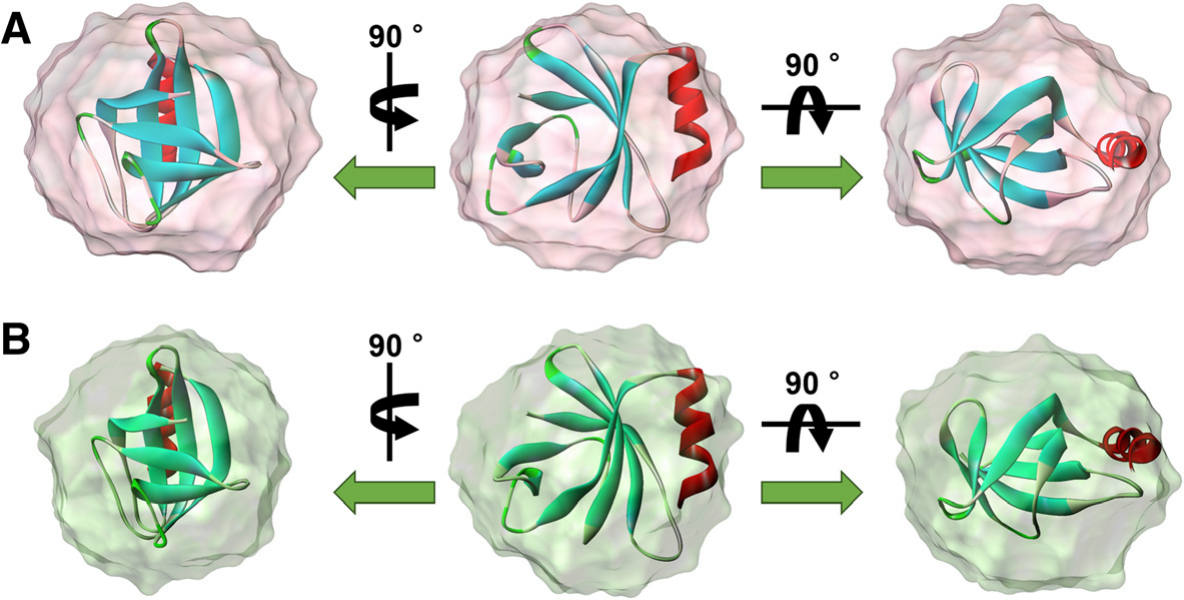
**Superimposition between the X-ray crystal structure and SAXS models of wild-type and mutant** ***E*** **.** ***coli*** **PmrD.** The superimposition was performed in SUPCOMB. The X-ray crystal structure is displayed as a ribbon diagram. **(A)** Wild-type PmrD. **(B)** Mutant PmrD.

To validate the usefulness of the SAXS models, the SUPCOMB superimposition program was used with template files such as the *ab initio* SAXS models and the crystal structure of *E. coli* PmrD. As shown in Figure 8, the superimposition models suggest that the PmrD crystal structure fits well to the *ab initio* SAXS models of the wild-type and dimer forms. The structural consentaneity between the crystal and modeled structures was substantiated quantitatively by means of normalized spatial discrepancy (NSD). The NSD algorithm facilitates rapid superimposition of structural models and offers useful information about the similarity between the models. It is expressed as the standard deviations for one-dimensional data sets. All NSD values between the crystal structure and the *ab initio* models were less than 0.6, implying that the reconstructed SAXS models were nearly identical to the crystal structure, and that the mutant PmrD structure was also akin to wild-type PmrD.

## Conclusions

In summary, the X-ray crystal structure of *E. coli* PmrD was determined at 2.00 Å resolution, showing that the structure comprised the C-terminal α-helix and the anti-parallel β-barrel containing six β-strands. Moreover, there is a distinguishing disulfide bond between the Cys9 residue in the β1 strand and the Cys81 residue in the α-helix, unlike for the other structures of PmrD in the crystal structure. Additional experiments offered more detailed characterization of the intra-disulfide bond. Native gel electrophoresis and SPR measurements showed that the removal of the disulfide bridge in PmrD did not influence the innate role of *E. coli* PmrD. On the other hand, the disruption of the internal bond resulted in increased overall size of PmrD, as demonstrated by size-exclusion chromatography and DLS. This structural alteration was further confirmed via *ab initio* SAXS modeling. Well-reconstructed SAXS models gave an abundance of information, including overall shapes and sizes.

The Groisman group has recently published new findings on discrepancies in the polymyxin B-resistance pathway among several bacteria [33]. They replaced the *E. coli pmrB* gene with the *Salmonella* homolog, resulting in the resistance of *E. coli* to polymyxin B under PmrD-inducing conditions. This result implies that disparities in the polymyxin B-resistance pathway among bacteria could stem from other factors, such as *pmrB*, rather than from structural differences between the PmrD proteins. Nevertheless, obvious reasons for the discrepancy have not been investigated; therefore, the structural analysis of PmrD is still significant. Our group has been continuously attempting to resolve the structures of PmrA and PmrB. It is anticipated that this investigation will provide critical insights into the bacterial signal-transduction mechanism.

## Availability of supporting data

The data sets supporting the results of this article are included within the article and its additional files.

## Additional files

Additional file 1: Table S1. Data and refinement statistics. Additional file 2: Figure S1. Size distributions of wild-type and mutant *E. coli* PmrD measured by DLS. Additional file 3: Figure S2. SAXS data. Experimental scattering curves of wild-type and mutant *E. coli* PmrD. The theoretical SAXS profile evaluated from the PmrD crystal structure [PDB: 4HN7] was also plotted. Additional file 4: Figure S3. Residuals of the scattering patterns of the crystal structure and SAXS data for: (A) wild-type PmrD. (B) mutant PmrD. Additional file 5: Figure S4. (A) Guinier plots for wild-type PmrD and mutant PmrD. The right table shows the slopes and *R*_g_,_G_ values for each plot. (B) Residuals of wild-type and mutant PmrD. Additional file 6: Figure S5. (A) Pair distance distribution function *p*(*r*) profile of wild-type and mutant *E. coli* PmrD using GNOM program. (B) Kratky plots for wild-type and mutant *E. coli* PmrD. Additional file 7: Table S2. Parameters calculated from the Guinier plots and pair distance distribution functions.

## Abbreviations

PmrD: Polymyxin B resistance protein D
*E. coli*: *Escherichia coli*
SPR: Surface plasmon resonance
DLS: Dynamic light scattering
SAXS: Small-angle X-ray scattering
LPS: Lipopolysaccharide
*S. enterica*: *Salmonella enterica*
TCSs: Two-component systems
*KP*: *Klebsiella pneumoniae*
IPTG: 1-thio-β-D-galactoside
CCD: Charge-coupled device
PAL: Pohang Accelerator Laboratory
PDB: Protein Data Bank
DCM: Double crystal monochromator
NSD: Normalized spatial discrepancy
PCR: Polymerase chain reaction
