# Supplementary material for: Structural insights into Escherichia coli polymyxin B resistance protein D with X-ray crystallography and small-angle X-ray scattering
Source: BMC Struct Biol. 2014 Dec 5;14:24. doi: 10.1186/s12900-014-0024-y (PMC4263063; doi:10.1186/s12900-014-0024-y)
Supplement: Additional file 1: Table S1. — Data and refinement statistics. [file 12900_2014_24_MOESM1_ESM.doc]

**Table S1**

Data and Refinement statistics

| **Data Statistics** | ***E*. *coli* PmrD** |
| --- | --- |
| Number of reflections (unique/total) | 5617/94856 |
| Resolution range (Å)^a^ | 50.0 – 2.0 (2.07 – 2.0) |
| Completeness (%)^a^ | 79.6 (28.1) |
| I/sigma I^a^ | 25.8 (21.2) |
| Redundancy^a^ | 16.9 (10.6) |
| R_merge_ (%)^a, b^ | 6.1 (48.5) |
| Unit cell parameter (Å) | a=83.580, b=83.580, c=75.951, α= β=90°, γ=120° |
| Space group | H32 |
| **Refinement Statistics** |  |
| Resolution range (Å) | 16.81 – 2.00 |
| Number of reflection (unique/total) | 4111/4306 |
| R/R_free_ (%)^c^ | 21.1/25.4 |
| No. of nonhydrogen atoms (protein/water) | 661/7 |
| **Model quality** |  |
| R.m.s. deviations |  |
| Bond lengths (Å) | 0.020 |
| Bond angles (°) | 2.160 |
| Average B factor (Å^2^) | 74.08 |
| Coordinate error (Å) | 0.066 |
| **Ramachandran statistics (%)** |  |
| Most favored | 95.2 |
| Additionally allowed | 4.8 |

^a^Values in parenthesis are for the highest resolution shell.

^b^R_merge_ = Σ|I ― <I>|/ Σ <I>, where I and <I> are the measured and averaged intensities of multiple measurements of the same reflection, respectively. The summation is over all the observed reflections.

^c^R= Σ|F_0_ ― F_c_|/ Σ|F_0_| calculated for all observed data. R_free_= Σ|F_0_ ― F_c_|/ Σ|F_0_|calculated for a specified number of randomly chosen reflections that were excluded from the refinement.
