# Supplementary figures and images for: Structural insights into Escherichia coli polymyxin B resistance protein D with X-ray crystallography and small-angle X-ray scattering
Source: BMC Struct Biol. 2014 Dec 5;14:24. doi: 10.1186/s12900-014-0024-y (PMC4263063; doi:10.1186/s12900-014-0024-y)

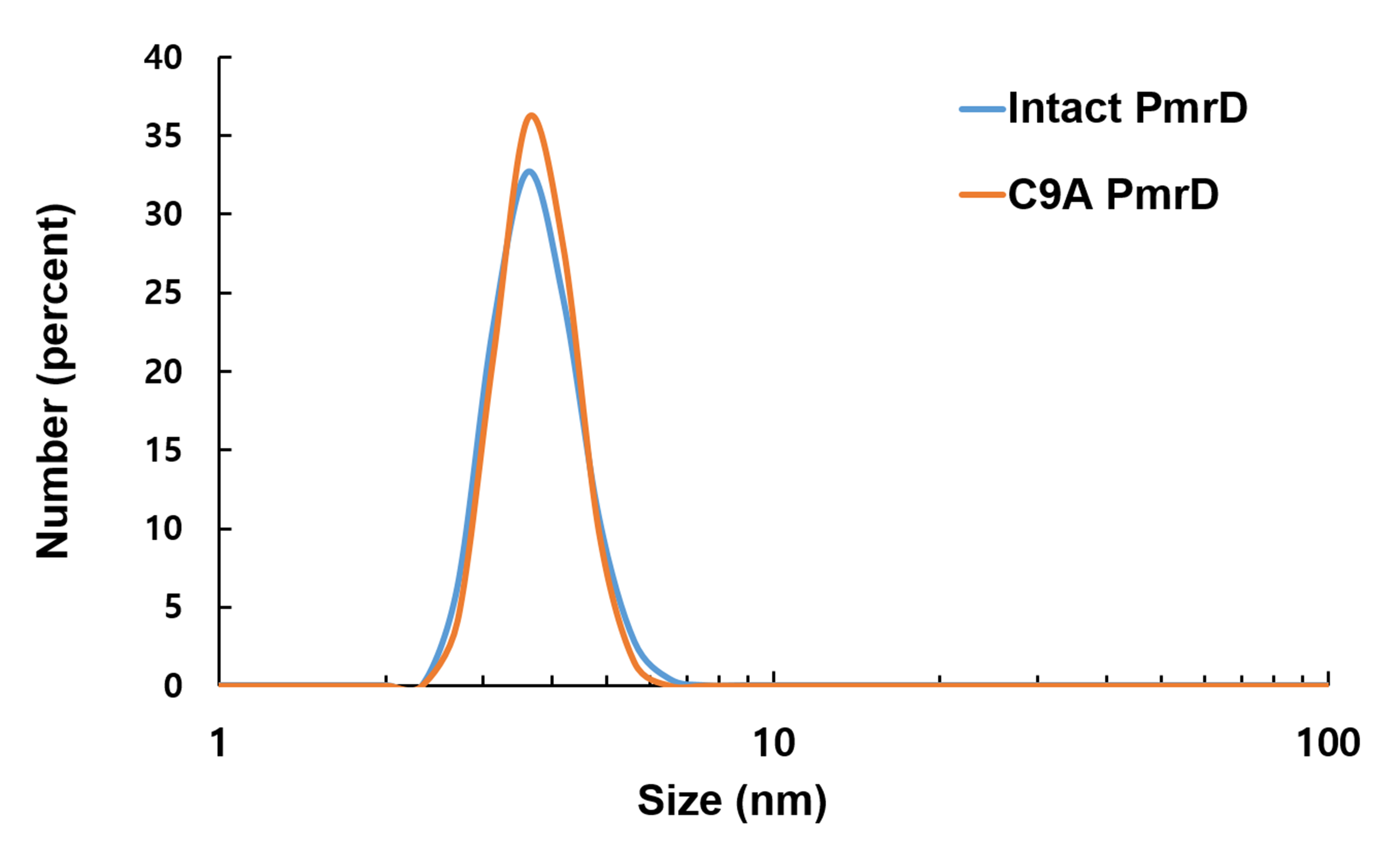

Supplement: Additional file 2: Figure S1. — Size distributions of wild-type and mutant E. coli PmrD measured by DLS. [file 12900_2014_24_MOESM2_ESM.tiff]

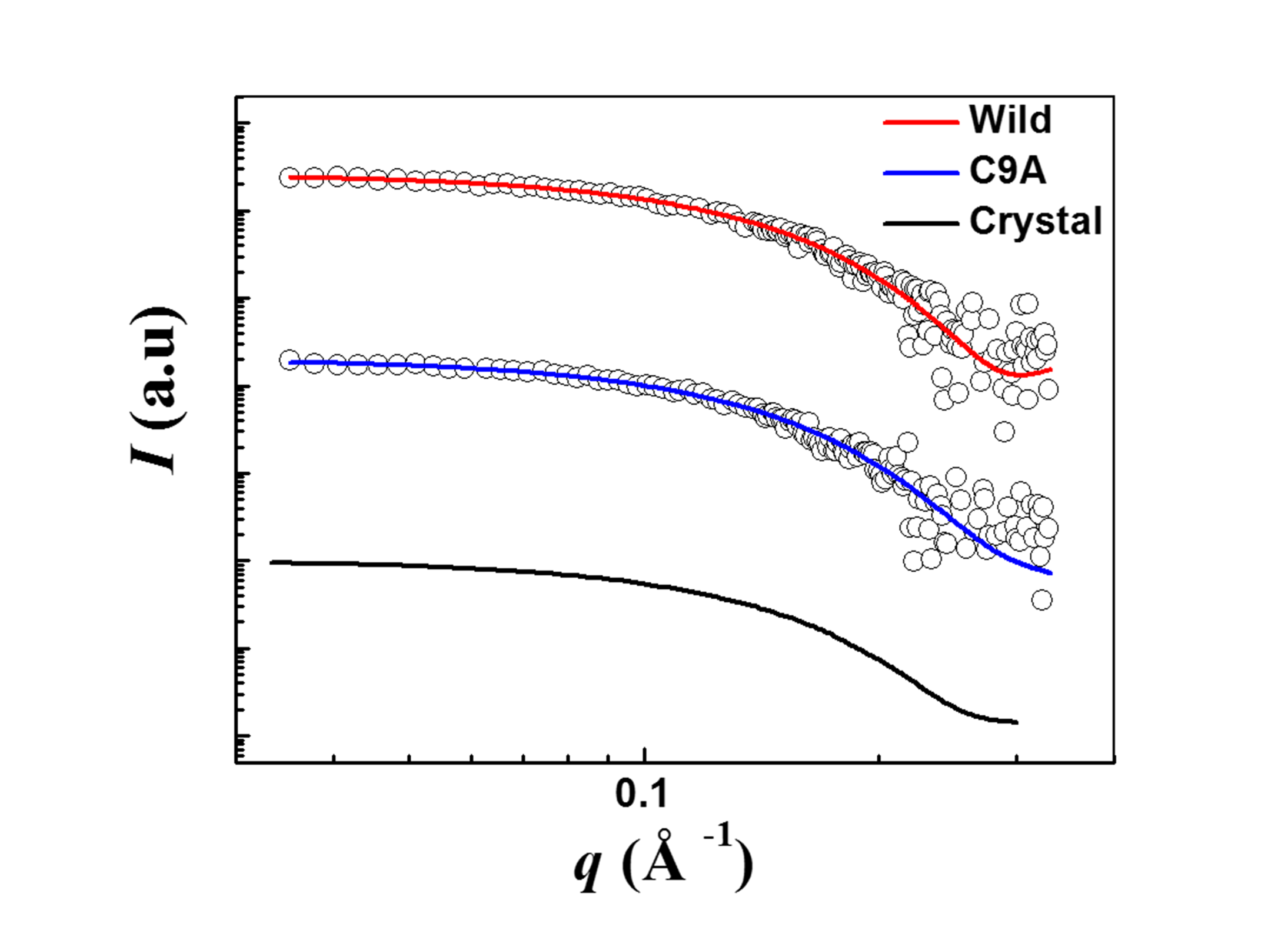

Supplement: Additional file 3: Figure S2. — SAXS data. Experimental scattering curves of wild-type and mutant E. coli PmrD. The theoretical SAXS profile evaluated from the PmrD crystal structure [PDB: 4HN7] was also plotted. [file 12900_2014_24_MOESM3_ESM.tiff]

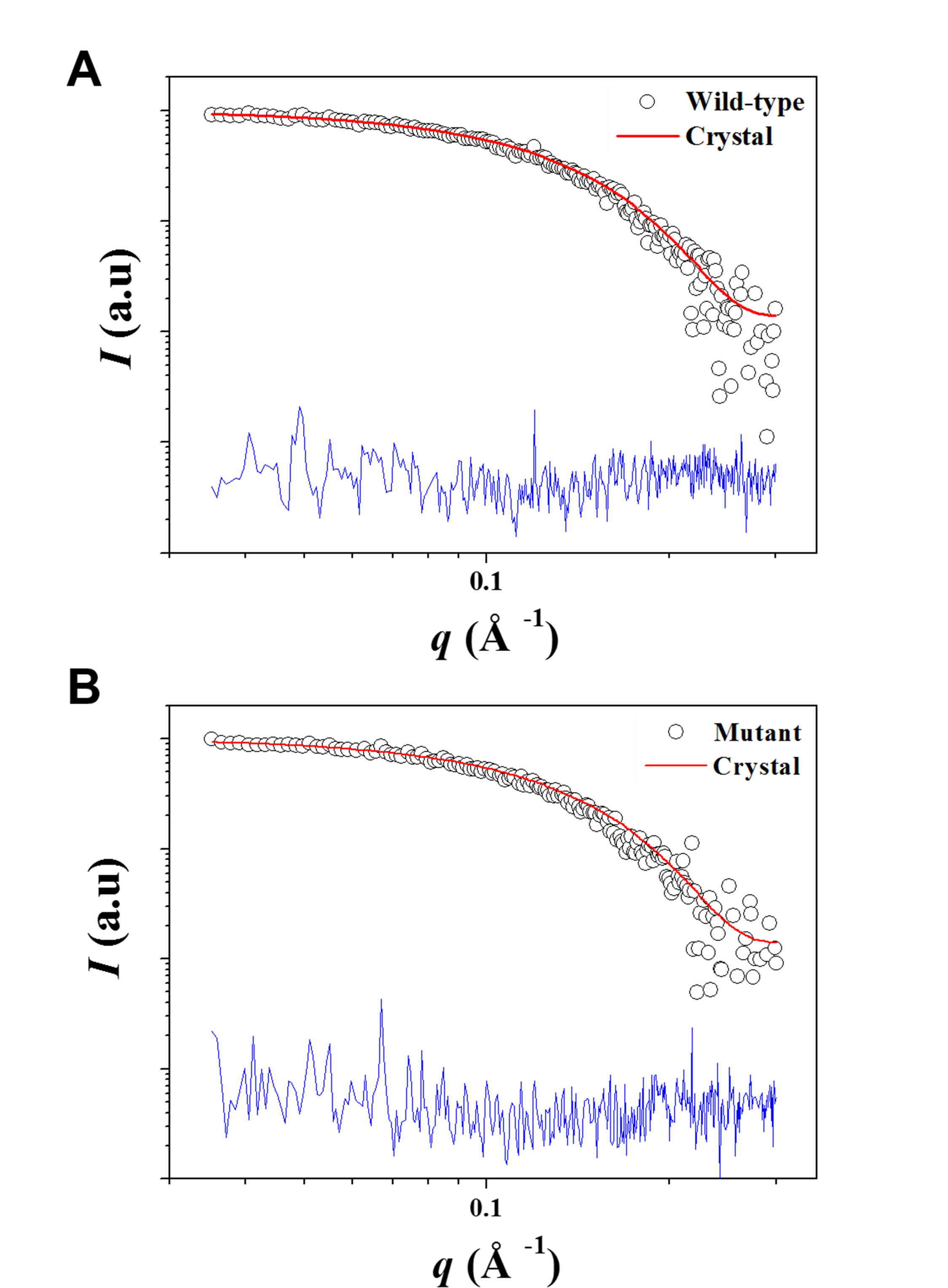

Supplement: Additional file 4: Figure S3. — Residuals of the scattering patterns of the crystal structure and SAXS data for: (A) wild-type PmrD. (B) mutant PmrD. [file 12900_2014_24_MOESM4_ESM.tiff]

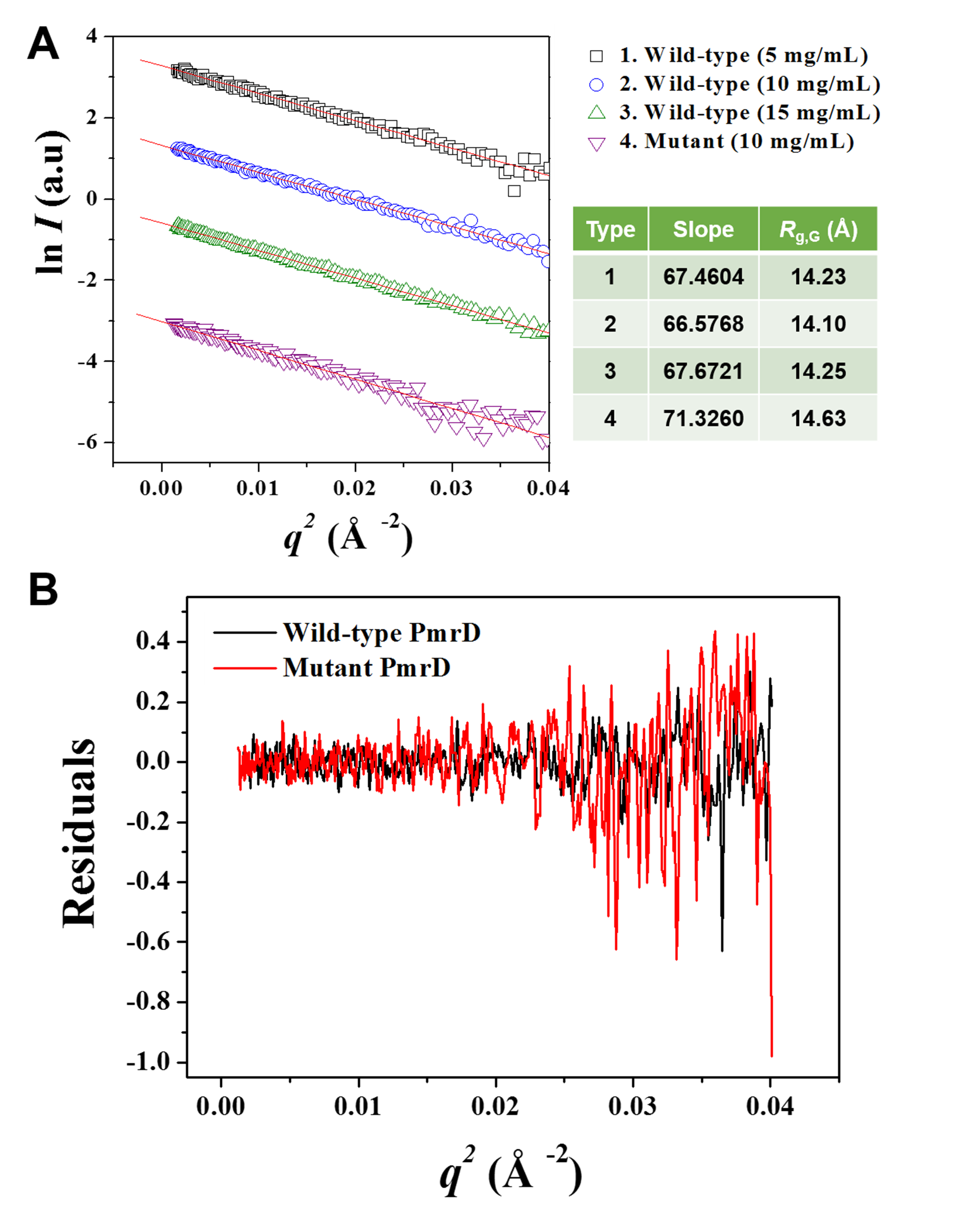

Supplement: Additional file 5: Figure S4. — (A) Guinier plots for wild-type PmrD and mutant PmrD. The right table shows the slopes and R g,G values for each plot. (B) Residuals of wild-type and mutant PmrD. [file 12900_2014_24_MOESM5_ESM.tiff]

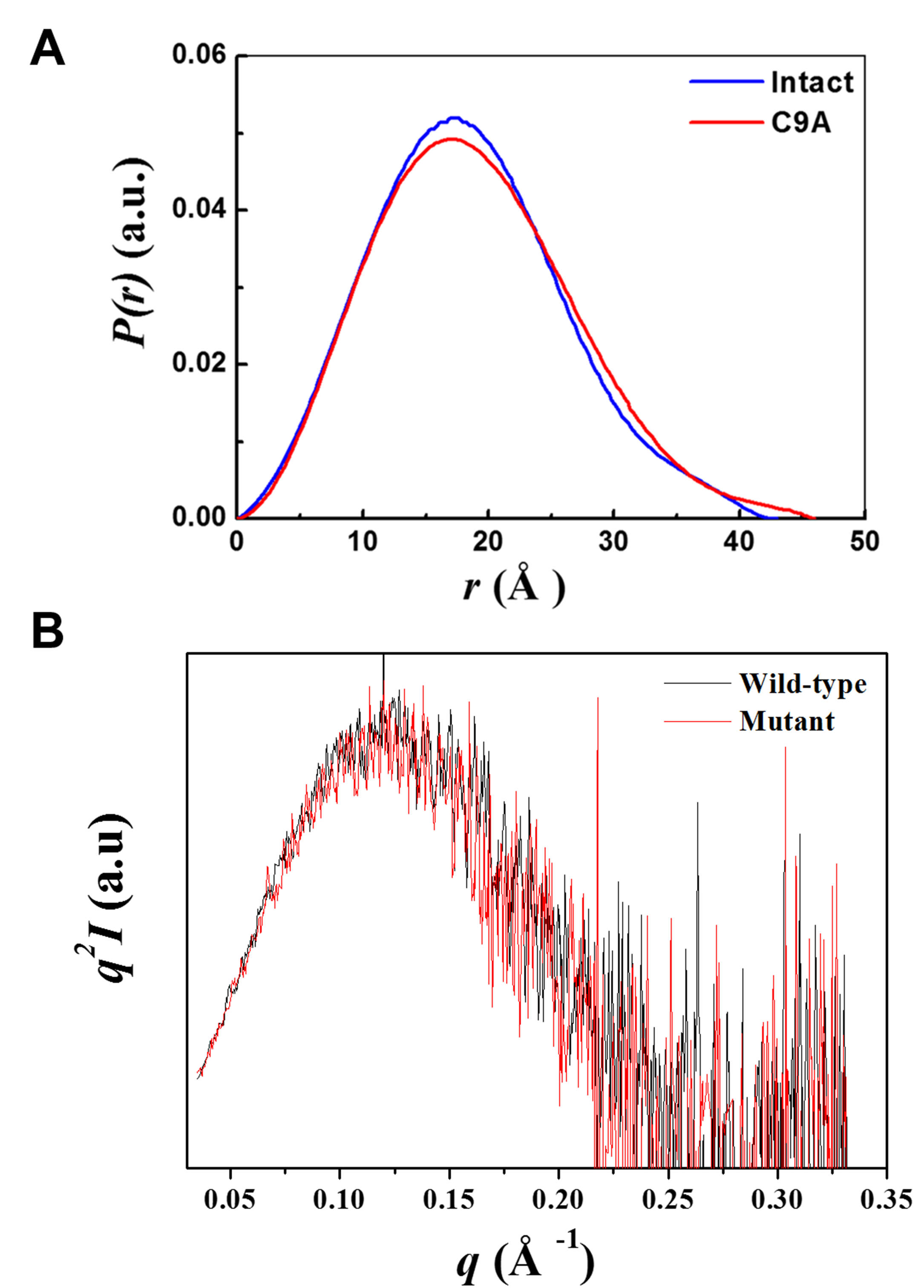

Supplement: Additional file 6: Figure S5. — (A) Pair distance distribution function p(r) profile of wild-type and mutant E. coli PmrD using GNOM program. (B) Kratky plots for wild-type and mutant E. coli PmrD. [file 12900_2014_24_MOESM6_ESM.tiff]
