# Supplementary material for: Structural insights into Escherichia coli polymyxin B resistance protein D with X-ray crystallography and small-angle X-ray scattering
Source: BMC Struct Biol. 2014 Dec 5;14:24. doi: 10.1186/s12900-014-0024-y (PMC4263063; doi:10.1186/s12900-014-0024-y)
Supplement: Additional file 7: Table S2. — Parameters calculated from the Guinier plots and pair distance distribution functions. [file 12900_2014_24_MOESM7_ESM.docx]

|  | *R*_g_,_G_ (Å)  (Guinier plot) | *R*_g_,*_p_*_(_*_r_*_)_ (Å)  (*p(r)* function) | I(0)  (Guinier plot) | I(0)  (*p(r)* function) | Porod- volume (Å^3^) | *D*_max_ (Å) |
| --- | --- | --- | --- | --- | --- | --- |
| Wild-type | 14.10 | 14.04 | 26.31 | 25.90 | 22036 | 43.1 |
| Mutant | 14.63 | 14.46 | 20.09 | 20.13 | 22098 | 46.0 |
